# Management of post burn hand deformities

**DOI:** 10.4103/0970-0358.70727

**Published:** 2010-09

**Authors:** S. Raja Sabapathy, Babu Bajantri, R. Ravindra Bharathi

**Affiliations:** Department of Plastic, Hand, Reconstructive Microsurgery and Burns Surgery, Ganga Hospital, 313, Mettupalayam Road, Coimbatore, Tamil Nadu, India

**Keywords:** Postburn hand deformity, contracture release, hand burns

## Abstract

The hand is ranked among the three most frequent sites of burns scar contracture deformity. One of the major determinants of the quality of life in burns survivors is the functionality of the hands. Burns deformities, although largely preventable, nevertheless do occur when appropriate treatment is not provided in the acute situation or when they are part of a major burns. Reconstructive procedures can greatly improve the function of the hands. Appropriate choice of procedures and timing of surgery followed by supervised physiotherapy can be a boon for a burns survivor.

## INTRODUCTION

**Figure 1:**
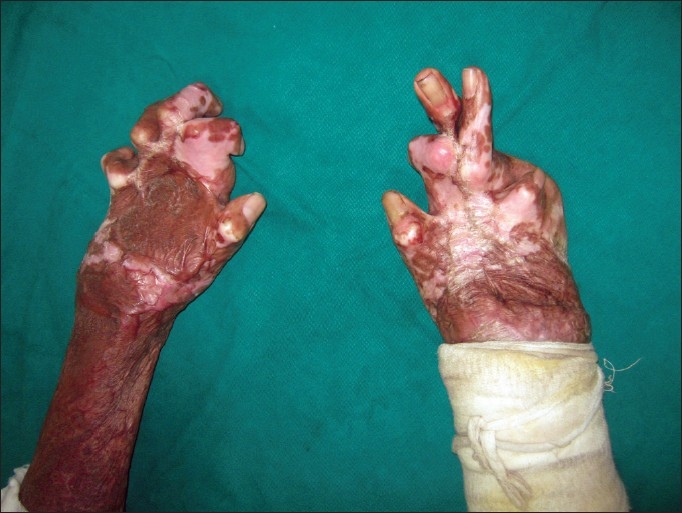
Severe burn contractures of both hands making this burn survivor a total cripple

One of the major determinants of the quality of life in burns survivors is the functionality of the hands. Postburn hand deformities, if bilateral, can make a burn survivor a total cripple [Figure 1]. The problem is largely preventable by good initial care, which would include elevation of the hand, appropriate splinting, early grafting of deep burns and supervised physiotherapy. Tredget[1] found that in patients with a mean total body surface area burn of 15%, 54% of the patients sustained burns to the hand and upper extremity. Because of the high frequency of occurrence of hand burns, the chances of the occurrence of a deformity is high. The hand is ranked as one of the three most frequent sites of burn scar contracture deformity.[2]

In this article, we have first explained the general principles that were found useful in treating burn hand deformities, followed by technical aspects pertaining to commonly seen deformities.

## GENERAL PRINCIPLES IN THE MANAGEMENT OF POSTBURN HAND DEFORMITIES

1. While reconstructing a burnt hand, the burn surgeon must concentrate on restoring function than just on increasing the range of movement of individual joints.
  Surgery on the burnt hand must restore pinch, the ability to grasp large objects and the power grip. This is obtained when the thumb pulp meets the pulp of other fingers, the hand has adequate first web space and the musculo tendinous units function to provide adequate power. Surgical procedures must be chosen to achieve these rather than aiming for an increase in the range of movement in each individual joint. For example, it might be an advantage to have a PIP joint arthrodesed in good functional position than to perform complicated procedures to restore movement in a bad boutonniere deformity.
2. When a hand is severely involved, choose the first set of procedures that will bring the maximum benefit to the patient.
  It is usual for a severe burn contracture to undergo a series of procedures to obtain the ultimate functional result, but the first procedure must produce a perceivable improvement in function. Early restoration of independence in the use of the hand will boost the morale and encourage the patient to adhere to postoperative protocols and take up subsequent procedures.
3. Function is very important, but a burn surgeon must also constantly think of the aesthetic aspect of reconstruction of a burned hand.
  The hand is a part that is always exposed and constantly reminds the patient that he is different. An aesthetically acceptable reconstruction helps him or her to easily integrate back into the society. The statement by Guy Foucher that “hand surgery is also aesthetic surgery” has never been truer than in the treatment of burned hands.[3]
4. Assess the deformity in each tissue component to make the treatment plan.
  Burn deformities occur secondary to skin loss. But, deformity correction involves not only correcting the skin loss but also the secondary changes that have occurred in the musculo tendinous units and joints. They usually are the limiting factors for deformity correction. Evaluate the deformity in each of the components of skin, tendons, joints and bones while making the treatment plan.
5. Correction of the deformity depends on the excision of the scar tissue and correcting the deforming forces than on the type of skin cover provided.
  Most deformity correction would need skin replacement. Mere replacement of the burn scar with skin graft or a flap will not correct the burn deformity.
6. Timing of surgery is crucial to get a good outcome in deformity correction. It is better to perform the surgery when there is tissue equilibrium, as shown by a reduction of the induration and the scars becoming pale.
7. Physiotherapy, splinting and scar control measures are important to achieve good outcome.

All these principles will apply to the correction of any burn deformity in some way or the other. Burn scar contractures have been classified by Mc Cauley[4] [Table 1].

**Table 1:** Classification of burn scar contracture

Grade I	Symptomatic tightness but no limitations in range of motion, normal architecture
Grade II	Mild decrease in range of motion without significant impact on activities of daily living, no distortion of normal architecture
Grade III	Functional deficit noted, with early changes in normal architecture of the hand
Grade IV	Loss of hand function with significant distortion of normal architecture of the hand
Subset classification for Grade III and Grade IV contractures: A: Flexion contractures, B: Extension contractures, C: Combination of flexion and extension contractures	

## MANAGEMENT OF INDIVIDUAL DEFORMITIES

### Dorsal hand deformities

**Figure 2:**
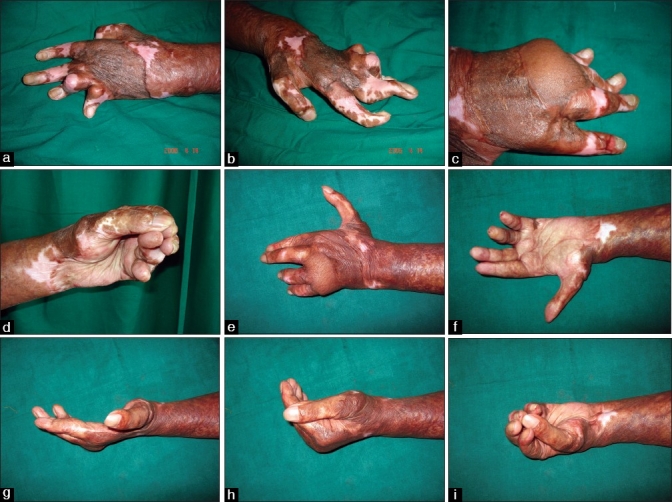
Dorsal burn contracture (a, b). The hand functionless due to narrow first-web space, and MCP joints stiff in extension with associated IP joint deformities (c). MP joints extension contracture corrected and flap given along with volar contracture release in the fingers. This procedure makes the hand functional (d). Arthrodesis of the IP joints in stages makes the hand aesthetically and functionally good (e-i)

In the classical dorsal hand deformity, there is clawing of the fingers – hyperextension of the metacarpophalangeal joints and flexion of the interphalangeal joints. [Figure 2]. The severe oedema in the dorsum of the hand causes hyperextension of the metacarpophalangeal joints. The palmar arches flatten. Flexion of the PIP joint occurs as a result of this oedema-imposed tension on the common digital extensor tendon system and concurrent hyperextension of the MP joints. The ring and the little fingers are affected 65% of the times.[5]

With time, the joints become stiff and the extensor tendons undergo adaptive shortening. The poor quality of skin at the PIP joint may expose the tendon and the extensor may rupture secondary to stretch and ischaemia. The thumb may lie in the plane of the palm with a narrow first web space.

The goal of treatment is to get the fingers straight, the MCP joints flexed and facilitate the thumb to oppose the tips of the other fingers. If the IP joints have fixed flexion deformity, they need to be released earlier to or along with the correction of the hyperextension deformity of the MCP joints. Otherwise, the fingertips will bury into the palm, functionally downgrading the hand. Adequate release of the IP joints that can be managed with skin grafts is performed if dorsal release is also attempted [Figure 2]. If, after the correction of the IP flexion deformity, the raw areas would need a flap, then dorsal release is not combined.

**Figure 3:**
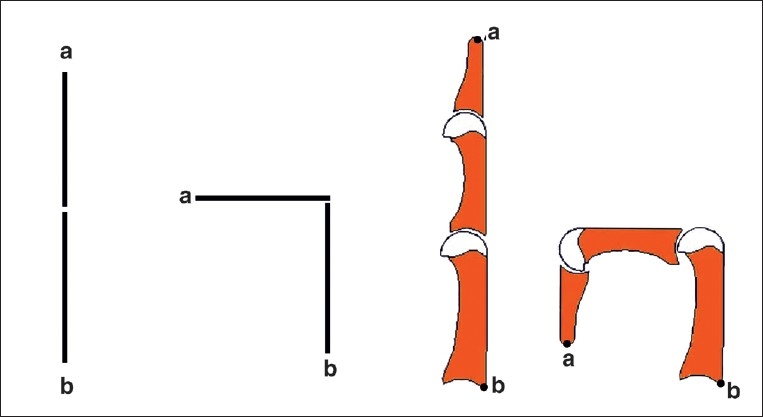
The flexion of the fingers is unlike that of the door hinge. There is considerable increase in length as the phalanges glide over the proximal bones, which require more skin after contracture release

Skin over the MCP and the IP joints are highly flexible, with reservoirs of skin overlying the joints. It has been documented that there is a considerable increase in finger length when moving from a position of total finger extension to complete fisting. The fingers do not move like a door hinge; rather, the phalanges articulate around the head of the antecedent segment to account for the increase in length [Figure 3]. This point has to be remembered when replacing skin after dorsal release.

**Figure 4:**
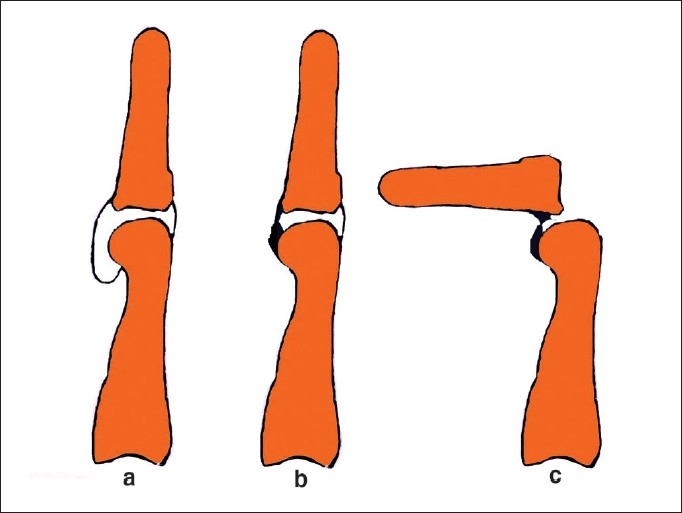
The anterior capsule allows the base of the proximal phalanx to glide over the head of the metacarpal (a). During contracture release, the adhesions over the head of the metacarpal (b) have to be released, otherwise the base will dislocate on attempting to correct the deformity (c)

Apart from skin shortage, two factors limit release of the contracture. One is the joint problem. The anterior capsule becomes stiff and adherent to the joint surfaces, preventing the proximal phalanx to glide volarwards [Figure 4]. On pressure, the base of the proximal phalanx may tilt upwards. When this happens, the capsule needs to be opened and, with the help of a curved dissector, a pocket needs to be created for the phalanx to glide forward.

**Figure 5:**
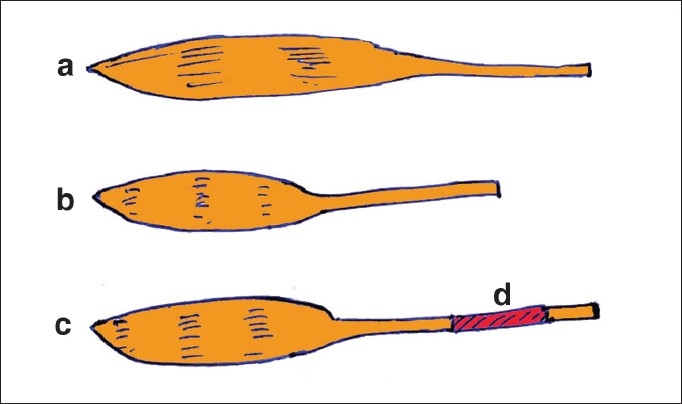
(a) Normal muscle. (b) Contracted muscle. (c) When a tendon graft (d) is used after contracture release, the muscle fibre length remains short and excursion of the muscle is reduced

The other factor is the adaptive shortening of the extensor tendons. It is better to stretch the muscles as much as possible, fix the joint and further stretch the contracted muscles by physiotherapy. Cutting the tendon and putting in a tendon graft is not recommended. Excursion of the tendon is dependent on muscle fibre length. When the gap is bridged by tendon graft, the muscle remains short and the length of excursion of the tendon becomes limited [Figure 5]. Sawhney feels that by staged release, skin grafting and rigorous physiotherapy, even very severe contractures can be corrected without resorting to flap cover and tendon grafting.[6] In very severe contractures, if the patient cannot afford the time for multiple procedures, the extensor tendons can be divided to obtain flexion of the fingers. Surprisingly, many patients adjust without extensor reconstruction.

**Figure 6:**
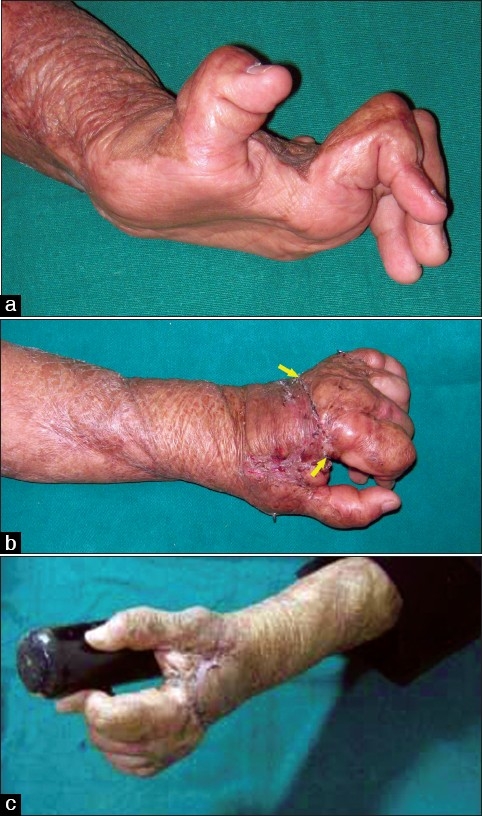
A severe dorsal contracture (a) Released by transverse incision placed proximal to the MCP joint (arrows). (b) The distal flap slides to cover the MCP joints, enabling capsulotomy to be performed with temporary stabilisation of the joints. This combined first-web release makes the hand functional (c)

**Figure 7:**
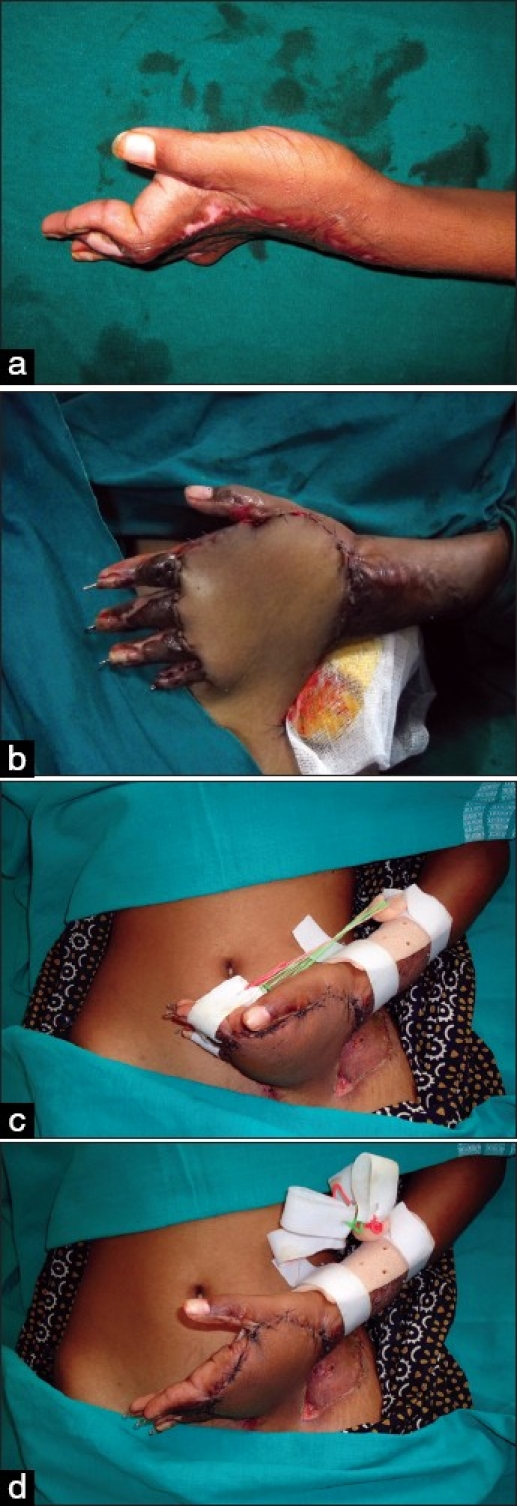
Severe dorsal and first-web contracture (a), corrected by contracture release and abdominal flap cover (b). At 10 days, dynamic exercises can be started even in the presence of pedical flaps (c, d)

The release is performed by a transverse incision placed in such a way that, after full release, the distal flap migrates and still covers the MCP joints [Figure 6]. Under the flap, MCP joint capsulotomy is performed. If the joint has to be temporarily stabilized, the K wires are to be obliquely inserted to avoid transfixing the extensor hood. Skin coverage is dependent on the bed and grafts are preferred if the bed would accept a graft or a flap is given. Skin flaps are preferable to muscle or fascial flaps with grafts because skin flaps allow easy access for secondary procedures. Whatever flap is used, the ultimate outcome is dependent on the physiotherapy to maintain the gains of surgery. While it is easier to institute early physio with free flaps, even with pedicled abdominal flaps as cover, it is possible to institute early physiotherapy [Figure 7].

### Volar deformities

Burns to the palm are less frequent when compared with dorsal burns and, more often, are superficial due to the thickness of the palmar skin. Deep burns of the palm occur in camphor burns associated with religious practices, industrial accidents and in electrical injuries. In the later two instances, hand deformities may be aggravated by the associated compartment syndrome and carpal tunnel syndrome. Early recognition of compartment syndrome and carpal tunnel syndrome and decompression will reduce deformities associated with palmar burns.

Established plamar contracture results in narrowing of the metacarpal arch with the thenar eminence approaching the hypothenar area. This results in hyperextension deformity of the MCP joint of the thumb and flexion at the IP joint.

**Figure 8:**
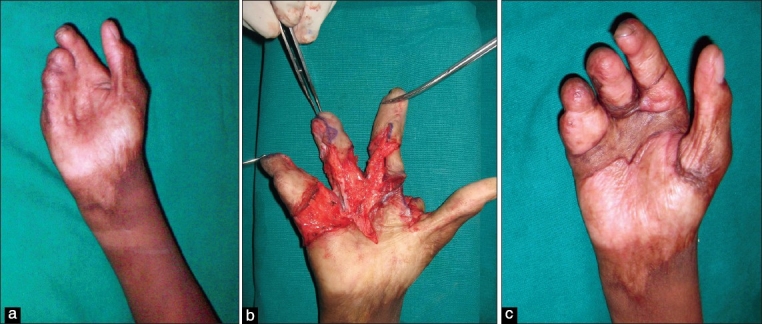
(a) Severe palmar contracture. (b) After release (c) Long-term maintenance with full-thickness grafts as cover

Full release of palmar contracture is usually possible and the raw area is covered by full-thickness or thick split-thickness grafts [Figure 8]. The first metacarpal may need to be fixed in extension till the grafts settle. It is better to use thick skin grafts in the palm than a flap. The bulkiness of the flap prevents cupping of the palm and the ability to hold objects. The patients feel like they are already holding something in their hand. Flaps, if they have to be used, are to be thin or have to undergo multiple secondary thinning procedures. Skin grafts, even if they have to be used in multiple stages, ultimately provide a better outcome as they help in holding objects better.

### Deformities of the thumb

**Figure 9:**
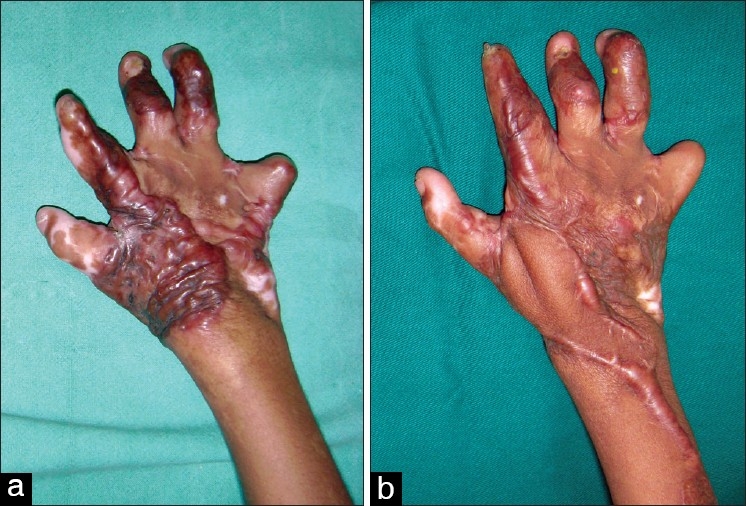
(a) Severe first-web contracture in a child corrected with posterior interosseous flap (b)

The most obvious deformity in the thumb appears at the MCP joint. When there is hyperextension contracture at the MCP joint, swan neck-type deformity occurs in the thumb and, with flexion contracture at the MCP joint, a boutonniere deformity occurs. Both may be associated with first-web contracture. When the deformities are of short duration and are due to skin contracture, release of the contractures will correct the problem. If the deformities are severe, an overall plan has to be made and it is linked with the deformities that the patient has in the other fingers. Adduction contractures of the first-web space can have a significant impact on overall hand function.[7] It might require release of the origin of the first dorsal interosseous from the first metacarpal and the adductor origin from the third metacarpal. Fibrous tissue overlying these muscles must also be released. In addition to gaining abduction, the aim is to obtain a certain amount of pronation of the thumb so that at the end of the contracture release, the pulp of the thumb will face the pulp of the other fingers. Release of the skin and first-web muscles will gain abduction, but pronation will be obtained only when the contracture at the CMC joint is released. This has to be done avoiding injury to the radial vessels. The raw area after first-web release almost always would need a flap cover. Posterior interosseous flap is an ideal choice if the donor site is available [Figure 9]. Otherwise, a distant pedicle flap or a free flap can be used.

At the same sitting, the thumb can be brought into a functional position by release of the contracture at the MCP joint. If difficult, an arthrodesis of the MCP joint of the thumb can be performed. The level and the inclination of bone freshening can be used to position the thumb in the most appropriate position. A good and adequate first-web and a stable MCP joint of the thumb will provide a functionally and aesthetically acceptable thumb in most instances.

### Swan neck deformities

A burn swan neck deformity occurs mainly due to three causes. They are, one: due to rupture of the extensor insertion at the distal interphalangeal joint causing a mallet deformity, which, secondarily, causes a swan neck deformity. A second cause is due to the skin contracture because of deep burns of the dorsum of the fingers. The third cause is due to tightening of the intrinsics due to ischaemic fibrosis of the intrinsic muscles secondary to compartment syndrome in the acute phase. Correction addressing the problem can only be effective if the deformity is not fixed. In acute trauma, the swan neck deformity secondary to a mallet deformity does not progress to a fixed deformity and, hence, correction of the mallet deformity corrects the swan neck deformity. In burns, most often, the extent of injury also extends proximally and the swan neck deformity quickly becomes a fixed deformity.

Swan neck deformity affects function, and it needs to be corrected. The choice will depend on the cause. If it is due to dorsal skin contracture, release incisions proximal and distal to the PIP joint and grafting will correct the deformity. Similarly, mallet deformity correction will correct it in the early stages. In all fixed deformities, the most viable option would be arthrodesis of the PIP joint in the functional position with increasing flexion from the index (25–30 degrees) to the little (40–50 degrees) fingers.[8]

### Boutonniere deformity

The deformity, characterized by flexion at the PIP joint and hyperextension at the DIP joint, is due to the rupture of the central slip insertion at the base of the middle phalanx. Tension ischaemia can result when the injured tendon is compressed between the eschar and the head of the proximal phalanx as the PIP joint is flexed.[9] The deformity could be prevented by splinting the PIP joint in extension in the acute stage. While it is advisable to encourage early movement in superficial burns, it is prudent to splint deep burns of the dorsum in the acute situation. If some movement is desired, it is better to advice isolated movement at each joint separately than a composite movement of all joints, which would put more stress on the injured tendons. In addition, pressure and tension on the tendons also causes ischaemic changes, promoting rupture of the tendon insertion.

Contrary to swan neck deformity, persons with boutonniere deformities continue to possess a fair amount of function. Surgery is indicated when the skin overlying the PIP joint is open, when there is severe hyperextension of the DIP or the MCP joints. Tendon-rebalancing procedures are rarely possible in burns because of the poor quality of the overlying skin, and arthrodesis is resorted to when there are problems of opposition to the thumb.

### Burn syndactyly

Circumferential burns of the fingers when allowed to heal without grafting cause syndactyly. The outcome of separation of burns syndactyly depends on the status and the depth of the burns of the other parts of the fingers. Usually, the IP joints are stiff and there is paucity of skin to make flaps, as is performed in congenital conditions. Most of the times, longitudinal division has to be made and the raw areas have to be grafted. This is the reason for the increased incidence of web creep after burn syndactyly separation than after congenital cases. When separating burn syndactyly, as far as possible, the best available skin is used to make the web flap. Full-thickness skin at the web prevents creep.

Web contractures are separated by using different types of Z plasty or by using the square flap technique. In burn contractures, in addition to deepening the web, fibrous tissue at the web also needs to be excised to promote lateral movement (abduction and adduction) of the fingers. Unless that is done, chances of recurrence are high. Postoperatively, padded splints are to be worn to prevent recurrence.

### The burnt little finger

The little finger is the most affected finger in burn deformities. Groenevelt, who performed a study on this, feels that little finger bears the brunt of burn trauma because it most exposed in the protective posture of the hand and, most often, suffers circumferential burns than other fingers.[10] The deformity could be a combination of flexion and rotation due to more loss of skin on the outer aspect of the finger. Initial management is to release the flexion and lateral contractures and get it out of the palm. If the finger is totally buried into the palm and if the other fingers can easily be restored to functional position, even amputation of the deformed little finger may be an advantage than undergoing multistage procedures with residual compromised function.

### Management of digital losses

Digital amputation may be an isolated digital loss like the thumb in case of electrical burns or multiple digital losses that occur in major burns. When the digital loss is an isolated one, the most valuable microsurgery procedure is toe transfer.[11,12] In large total body surface area burns with involvement of the feet and multiple digital amputations, free toe to hand transfers may not be possible. In them, finger lengthening techniques by distraction osteosynthesis are useful.[13] Corticotomy of the shortened digit is performed after placement of a mini-Hoffman device for subsequent distraction. After 2 weeks, early callus formation occurs with immature woven bone and osteoprogenitor cells. Subsequent distraction of the healing callus can occur followed by a 2–4-week period of stabilisation to allow the new bone to solidify. Distraction lengthening is mostly performed in the thumb and the index to restore pinch. For it to be effective, at least most of the first metacarpal must be present and skin coverage of the amputation stump should be adequate. Traditional techniques like phalangisation and osteoplastic thumb reconstruction are also rarely used.[14,15]

### Physiotherapy and aftercare of postburn hand deformities

The gains made by surgery have to be maintained by properly splinting the released joints. If Kirshner wires have been used to hold the joints in position, they are retained for 3 weeks to enable the grafts or flaps to settle. The grafts must be massaged and custom-made compression garments worn for 6 months for the grafts and flaps to settle. The scars have a grater tendency to contract in the early period, relentlessly acting throughout the day. Therefore, therapy has to be performed multiple times a day and the joints have to be splinted in between. Good physiotherapy will make all the efforts worthwhile.
